# Two new species of the genus *Dryopomorphus* Hinton, 1936 from China (Coleoptera, Elmidae)

**DOI:** 10.3897/zookeys.765.24366

**Published:** 2018-06-06

**Authors:** Dongju Bian, Xue Dong, Yunfei Peng

**Affiliations:** 1 CAS Key Laboratory of Forest Ecology and Management, Institute of Applied Ecology, Shenyang 110016, China; 2 Graduate University of Chinese Academy of Sciences, Beijing 100039, China

**Keywords:** *Dryopomorphus*, Guangdong, Riffle beetle, taxonomy, Yunnan

## Abstract

Two new species of the genus *Dryopomorphus* Hinton, 1936 are described from China: *Dryopomorphus heineri* **sp. n.** and *D. ruiliensis* **sp. n.** Habitus photographs and detailed line drawings of the male genitalia are provided.

## Introduction

The genus *Dryopomorphus* Hinton, 1936 occurs in eastern Asia, from China and Japan to Malaysia and Brunei. Fourteen species of this genus were described until now (Hinton 1971, Spangler 1985, Kodada 1993, Yoshitomi and Satô 2005, Čiampor and Kodada 2006, Čiampor et al. 2012, Yoshitomi and Jeng 2013, Jäch et al. 2016). Yoshitomi and Satô (2005) reviewed the four species of Japan. Čiampor et al. (2012) reviewed the *Dryopomorphus* species of Malaysia, from where three known species were diagnosed and five new species were described.

The presence of the genus *Dryopomorphus* in China was indicated by Jäch and Kodada (1995) based on specimens collected during the China Water Beetle Survey (CWBS), but the authors did not give detailed species information. In the catalogue of Elmidae (Jäch et al. 2016), there is no record of this genus from China, so this is the first time that the genus *Dryopomorphus* is reported from China formally. In this article, two new species of this genus, collected during the China Water Beetle Survey, are described.

## Materials and methods

Specimens were examined with a Leica M205c stereomicroscope and an Olympus BX51 compound microscope. Genitalia were drawn with the aid of a drawing tube. Male genitalia were placed in concentrated lactic acid in a cavity slide for at least several hours before they were examined. Habitus photographs were made with a KEYENCE VHX-2000 – Super Resolution Digital Microscope System. Label data are cited verbatim, with separate lines on the same label indicated by a slash “/”; different labels are separated by a vertical line“|”.

Abbreviations used in the text:

**BL** body length = PL+EL,

**BW** maximum width of body,

**PL** pronotal length,

**PW** maximum width of pronotum.

**EL** elytral length,

**EW** maximum width of elytra,

**CWBS** China Water Beetle Survey,

The type specimens of the new species are deposited in the Institute of Applied Ecology, Chinese Academy of Sciences, Shenyang, China (**IAECAS**) and in the Natural History Museum Vienna, Austria (**NMW**).

## Taxonomy

### Dryopomorphus heineri sp. n.

Taxon classificationAnimaliaColeopteraElmidae

http://zoobank.org/A1F823DD-1B47-42F6-BED0-17172409D63F

Figures 1–2 , 5–7

#### Type material.

Holotype, male (IAECAS): “CHINA: Guangdong, Yunfu, / Yu’nan, Tongle Mountains, | 23°11'49"N, 111°23'6"E, 278m, / 2017.11.16, Leg. Peng & Sun (10)”; Paratypes: 1 male (IAECAS), same data as holotype; 1 male (IAECAS): “CHINA: Guangdong, Maoming, / 22°16'22"N, 111°14'29"E, | 994m 2017.11.19 / Leg. Peng & Sun (17)”; 2 exs. (NMW): “CHINA: Guangdong Prov. / 38 km ENE Zengcheng / 23°16'37’’N, 114°03'19’’E / 11.11.2001, ca. 200 m / Komarek & Wang (CWBS 489) ”; 1 ex. (NMW): “CHINA: Guangdong Prov. / 45 km N Zengcheng / 23°37'28’’N, 113°50'10’’E / 13.11.2001, ca. 500 m / leg. M. Wang (CWBS 495)”.

#### Diagnosis.

Body elongate suboval, distinctly convex, large (BL: 3.64–3.81 mm), head and pronotum dark brown, elytra brown; anterior angles of pronotum explanate, distinctly protruding, median sulcus of pronotum clearly impressed from basal 0.1 to 0.6, sublateral sulci present in basal 0.4; elytral intervals 3, 5, 7 slightly elevated in basal third. This species is similar to *D. siamensis* Kodada, 1993 by parameres being angulate at apex, but it can be distinguished by its smaller and slimmer penis, which is distinctly shorter than the parameres.

#### Description

**Figures 1–4.**
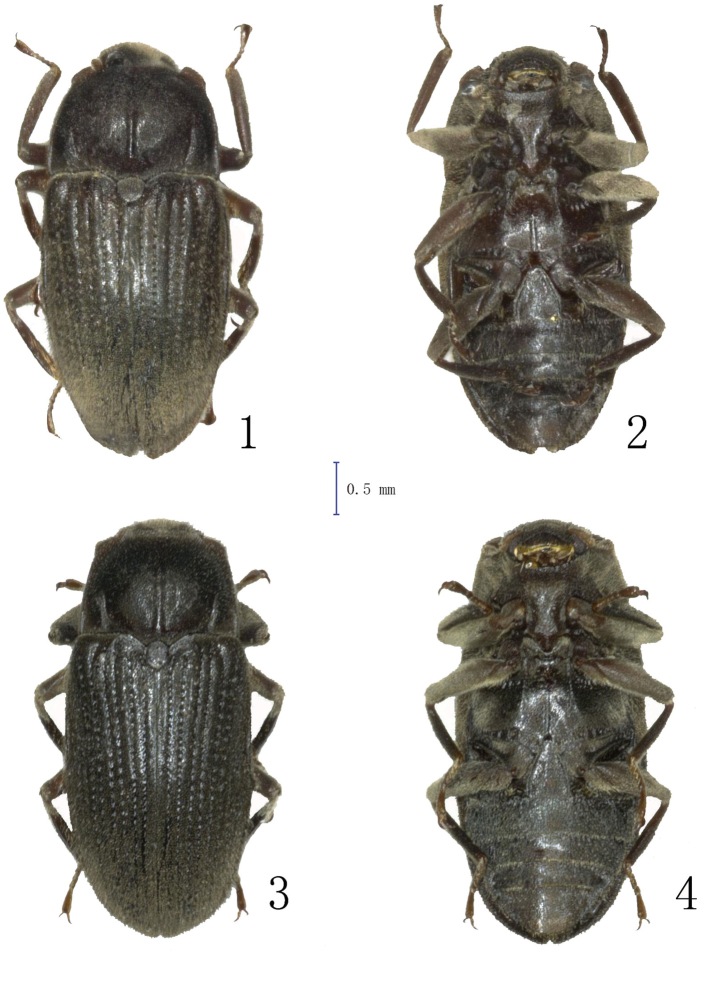
Habitus photographs.**1–2** *Dryopomorphus heineri* sp. n., holotype **3–4** *Dryopomorphus ruiliensis* sp. n., holotype **1, 3** in dorsal view **2, 4** in ventral view.

(holotype). Body elongate oval, BL 3.64 mm, BW 1.71 mm, distinctly convex dorsally, for habitus see figures 1–2, dorsal surface with dense short adpressed yellowish setae and sparse long semi-erect setae. Head and pronotum dark brown, elytra brown, antennae yellowish brown, frons and all legs reddish brown.

*Head*. Labrum short, partly concealed by clypeus, anterior margin truncate with dense yellowish long setae laterally. Clypeus approx. twice as wide as long, slightly emarginated anteriorly, densely punctate and pubescent. Frontoclypeal suture straight, distinct, but not deeply impressed. Vertex densely punctate, setose, with dark triangular area. Eyes large, slightly protruding from head outline, sub-triangular in lateral view.

*Thorax*. Pronotum broadest at base, PL 0.99 mm, PW 1.56 mm. Disc convex, punctures densely impressed on entire surface, lateral margin narrowly ridged, slightly converging anteriorly, anterior angles explanate, distinctly protruding. Median sulcus well impressed from basal 0.1 to 0.6, sublateral sulci present in basal 0.4. Prosternal process approx. 1.7 times as long as prosternum in front of coxae, lateral margin raised, distal 0.4 distinctly narrowed, ending with narrowly rounded apex, a pair of lateral protuberances at basal 0.6, surface covered with dense long setae. Mesoventrite distinctly constricted between coxae. Metaventrite with anterior 1/3 impressed between coxae; discrimen thin, present along entire length.

*Elytra*. EL 2.71 mm, EW 1.71 mm, distinctly convex, basal 1/2 subparallel, then converging to separately rounded apices, densely punctate and pubescent. Each elytron with 10 striae, punctures in discal part of striae medium-sized and in lateral part of striae larger and distinct; intervals 3, 5 and 7 slightly elevated in basal third, with dense micro-punctures. Scutellum longer than wide, sides arcuate.

*Abdomen.* Intercoxal process of first ventrite subtriangular with rounded apex, wider than long, lateral margin raised continuously into carina reaching hind margin of ventrite 1; admedian cavities short, oblique. Posterolateral angles of ventrites 2 to 4 distinctly protruding posteriorly. Apex of ventrite 5 emarginate medially.

**Figures 5–10.**
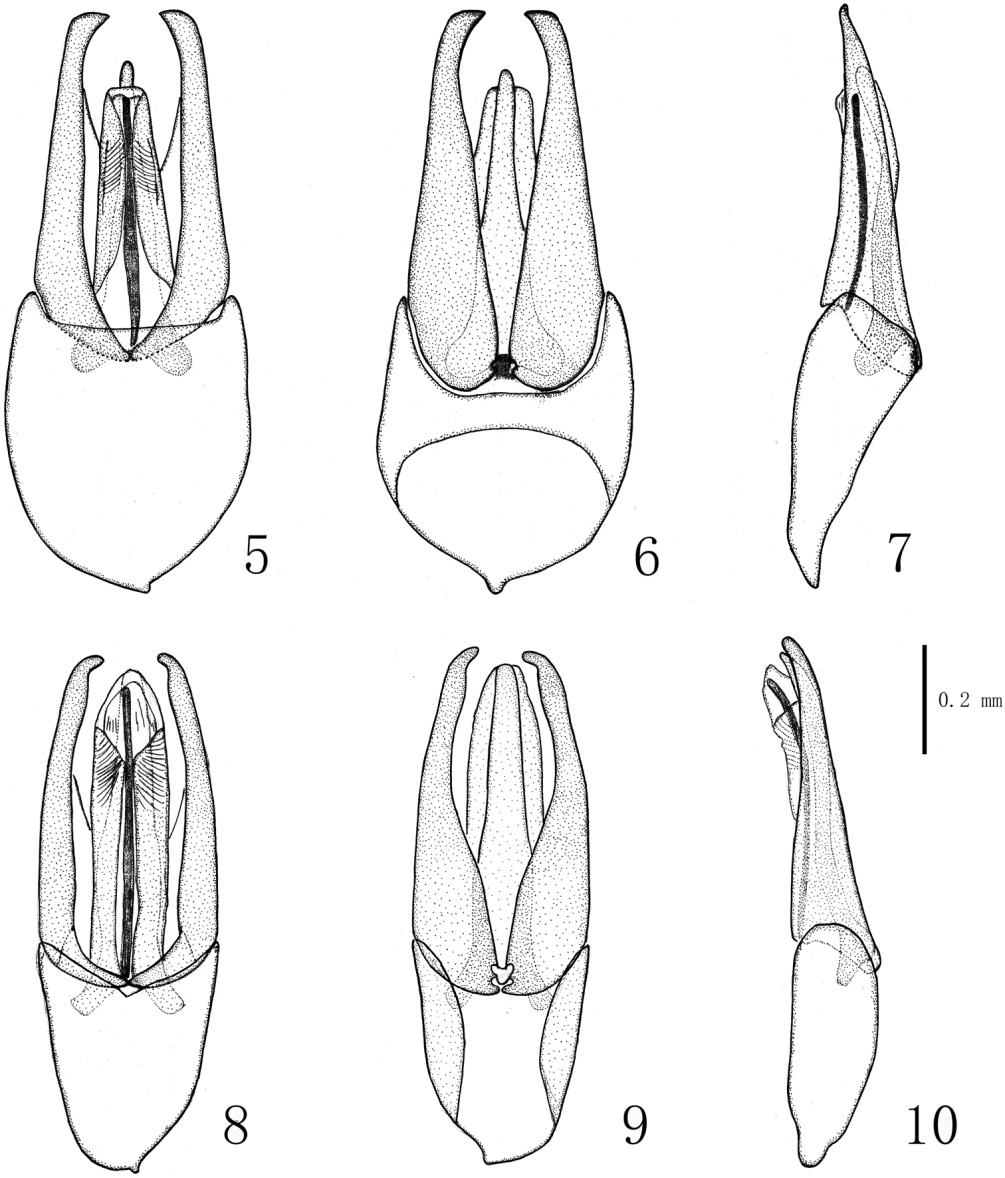
Male genitalia. **5–7** *Dryopomorphus heineri* sp. n. **8–10** *Dryopomorphus ruiliensis* sp. n. **5, 8** in ventral view **6, 9** in dorsal view **7, 10** in lateral view.

*Aedeagus* (figures 5–7). Approximately 1 mm long. In ventral view, phallobase wide, slightly shorter than penis, distinctly shorter than parameres. Distal half of penis distinctly narrowed, apices narrowly rounded; ventral sac folded, but distinctly expanding if placed in lactic acid for more than two days. Parameres distinctly longer than penis, gradually narrowed from base towards angulate apex.

Males: 3.64–3.81 mm long, 1.71–1.86 mm wide.

Females: unknown.

#### Distribution.

China (Guangdong).

#### Etymology.

This species is dedicated to our deceased friend Heinrich (Heiner) Schönmann, Vienna, a specialist in Hydrophilidae and good collector.

### Dryopomorphus ruiliensis sp. n.

Taxon classificationAnimaliaColeopteraElmidae

http://zoobank.org/C6F85333-3817-4B4F-B6C1-3976BD6BAD09

Figures 3–4 , 8–10

#### Type material.

Holotype, male (IAECAS): “CHINA: Yunnan, Ruili, / Yingjiang, ca. 830 m, | 24°6'35.634"N, 98°0'8.160"E, / 2016.10.17, Leg. Wang et al. (5)”. Paratypes: 2 males (IAECAS), 2 females (IAECAS), same data as holotype; 1 male (IAECAS): “CHINA: Yunnan, Yingjiang, / Nabang, 1308m, | 24°36'50"N, 97°35'58"E, / 2016.10.21, Leg. Peng & Sun (14)”; 1 female (IAECAS): “CHINA: Yunnan, Tengchong, / Mingguang, 1826m, | 25°28'39"N, 98°31'53"E / 2016.10.24, Leg. Peng & Sun (19)”; 1 ex. (NMW): “CHINA: Yünnan, Xishuangbanna / ca. 15km W Menglun / 5.11.1999, ca. 700–800 m / leg. Jäch, et al. (CWBS 354)”; 1 ex. (NMW): “CHINA: Yünnan, Xishuangbanna / ca. 10km NW Menglun / 7.11.1999, ca. 700 m / leg. Jäch, et al. (CWBS 359)”; 1 ex. (NMW): “CHINA: Yünnan, Xishuangbanna / ca. 10km NW Menglun / 7.11.1999, ca. 700–800 m / leg. Jäch, et al. (CWBS 360)”; 7 exs. (NMW): “CHINA: Yünnan, Xishuangbanna / ca. 6km NW Mengla / 8.11.1999, ca. 700 m / leg. Jäch, et al. (CWBS 365)”; 2 exs. (NMW): “CHINA: Yünnan, Xishuangbanna / ca. 6km NW Mengla / 9.11.1999, ca. 700 m / leg. Jäch, et al. (CWBS 367)”; 2 exs. (NMW): “CHINA: Yünnan, Xishuangbanna / pass betw. Jinghong – Mengyang / 12.11.1999, ca. 1100 m / leg. Jäch, et al. (CWBS 379)”.

#### Diagnosis.

Body elongate, dark brown; anterior angles of pronotum explanate, slightly protruding, median sulcus of pronotum straight, present at posterior 0.7 and sublateral sulci present in posterior 0.3; elytral punctures on disc small and in lateral striae large and distinct. This species can be distinguished from *Dryopomorphus heineri* sp. n. by: 1. pronotum less convex, anterior angles less protruding; 2. elytra subparallel in basal 2/3, then gradually converging posteriorly to separately rounded apices; 3. parameres slightly extending beyond penis, with apices narrowly rounded, whereas in *D. heineri* sp. n. the parameres distinctly extend beyond penis and the apices are angulate. This species can be distinguished from all other known species of the genus by the different shape of the parameres which are gradually narrowed from the base towards the apex in ventral view, distinctly narrowed in basal half in dorsal view, with sub-distal portion slightly inflated and apices narrowly rounded.

#### Description

(holotype). Body elongate, BL 4.14 mm, BW 1.88 mm, dorsal surface covered with short adpressed yellowish setae and long sub-erect setae which are black on the basal 3/4 and yellowish at the distal 1/4. Dorsum dark brown, anterior corners of pronotum and legs reddish brown, antennae yellowish brown, ventral surface dark brown.

*Head.* Partly retractable into thorax. Labrum short, densely setose, anterior margin almost truncate, with long yellowish setae, lateral angles almost rounded. Clypeus longer and wider than labrum, approx. twice as wide as long, densely setose, frontoclypeal suture straight; surface of labrum and clypeus densely punctate. Eyes large, slightly protruding from head outline. Pubescence of frons denser on a central triangular area; frons and vertex densely punctate, punctures setose.

*Thorax*. Pronotum widest at base, broader than long. PL 0.96 mm, PW 1.49 mm. Disc convex, densely punctate, lateral margin narrowly rimmed, slightly tilt inward, anterior angles slightly protruding. Median sulcus straight, present along posterior 0.7 of pronotum; sublateral sulci deeply impressed, present on posterior 0.3 of pronotum. Hypomeron sub-parallel, narrowed posteriorly, anterior depression developed for reception of antennae; surface coarsely punctate, densely setose. Prosternal process 1.8 times as long as prosternum in front of coxae, lateral margin distinctly raised and sub-parallel at anterior 0.7, posterior margin distinctly produced medially; surface of prosternum densely setose and sublateral area very coarse. Mesoventrite short, mesoventral cavity for reception of prosternal process very deep. Metaventrite impressed anteriorly between mesocoxae; discrimen narrow, present from anterior impression to posterior margin; disc flat, finely and densely punctate, densely setose; sub-lateral area with two kinds of punctures, fine punctures dense, large punctures sparse.

*Elytra* oblong, EL 3.18 mm, EW 1.88 mm; disc convex, densely pubescent, subparallel in basal 2/3, then gradually converging posteriorly to separately rounded apices. Each elytron with 10 striae; punctures deeply impressed, on disc and distal striae smaller, on lateral striae large and distinct. Intervals between striae slightly convex, with dense micro-punctures. Scutellum longer than wide, subpentagonal.

*Abdomen*. Intercoxal process of the first ventrite triangular, longer than wide, lateral margin with carina reaching posterior margin of first ventrite, admedian cavities short, oblique. Lateral portion of ventrites 1–3 with large punctures; posterolateral angles of ventrites 2–4 protruding posteriorly. Apex of ventrite 5 rounded. Surface of ventrites finely and densely punctate, densely pubescent.

*Aedeagus* (figures 8–10). Approximately 0.9 mm. In ventral view (figure 8), phallobase distinctly shorter than penis; parameres gradually narrowed from base towards apex, slightly extending beyond penis, sub-distal portion slightly inflated, apices narrowly rounded; penis robust, ventral sac finely sculptured in apical portion. In dorsal view (figure 9), parameres distinctly narrowed in basal half.

Males: BL 3.93–4.14 mm, BW 1.80–1.88 mm.

Females: BL 4.04–4.08 mm, BW 1.82–1.88 mm. Externally similar to males.

#### Distribution.

China: Yunnan.

#### Etymology.

This species is named after Ruili City, Yunnan Province, China.

#### Habitat.

A photograph of the habitat of *D. ruiliensis* sp. n. was published by Jäch and Ji (2003: figure 5).

## Supplementary Material

XML Treatment for Dryopomorphus heineri

XML Treatment for Dryopomorphus ruiliensis
