# Corrigendum: Two new species of the genus *Dryopomorphus* Hinton, 1936 from China (Coleoptera, Elmidae). ZooKeys 765: 51–58. (2018). https://doi.org/10.3897/zookeys.765.24366

**DOI:** 10.3897/zookeys.855.36298

**Published:** 2019-06-13

**Authors:** Dongju Bian, Xue Dong, Yunfei Peng

**Affiliations:** 1 CAS Key Laboratory of Forest Ecology and Management, Institute of Applied Ecology, Shenyang 110016, China CAS Key Laboratory of Forest Ecology and Management, Institute of Applied Ecology Shenyang China; 2 University of Chinese Academy of Sciences, Beijing, 100049, China University of Chinese Academy of Sciences Beijing China

## Abstract

none

One school name needs to be corrected:

**Page 51**:

**1***CAS Key Laboratory of Forest Ecology and Management, Institute of Applied Ecology, Shenyang, 110016, China*.

**2***Graduate University of Chinese Academy of Sciences, Beijing, 100039, China*.

**The correct one should be**:

**1***CAS Key Laboratory of Forest Ecology and Management, Institute of Applied Ecology, Shenyang, 110016, China*.

**2***University of Chinese Academy of Sciences, Beijing, 100049, China*.

